# Liquid Guayule Natural Rubber, a Sustainable Processing Aid, Enhances the Processability, Durability and Dynamic Mechanical Properties of Rubber Composites

**DOI:** 10.3390/ma15103605

**Published:** 2022-05-18

**Authors:** Xianjie Ren, Cindy S. Barrera, Janice L. Tardiff, Andres Gil, Katrina Cornish

**Affiliations:** 1 Department of Food, Agricultural and Biological Engineering, Ohio Agricultural Research and Development Center, The Ohio State University, 1680 Madison Avenue, Wooster, OH 44691, USA; ren.447@osu.edu; 2 Research and Advanced Engineering, Ford Motor Company, 2101 Village Road, Dearborn, MI 48124, USA; cbarre61@ford.com (C.S.B.); jtardiff@ford.com (J.L.T.); 3 Applications Engineering, Alpha-Technologies, Hudson, OH 44106, USA; andres.gil@alpha-technologies.com; 4 Department of Horticulture and Crop Science, Ohio Agricultural Research and Development Center, The Ohio State University, Williams Hall, 1680 Madison Avenue, Wooster, OH 44691, USA

**Keywords:** processing aid, carbon black, dynamic mechanical properties, damping performance, oil resistance

## Abstract

Petroleum-based oils are widely used as processing aids in rubber composites to improve processability but can adversely affect rubber composite performance and increase carbon footprint. In this research, liquid guayule natural rubber (LGNR), produced from guayule natural rubber, was used as a renewable processing aid to replace naphthenic oil (NO) in Hevea natural rubber, styrene-butadiene rubber (SBR) and guayule natural rubber (GNR) composites. The rheological properties, thermal stability, glass transition temperature, dynamic mechanical properties, aging, and ozone resistance of rubber composites with and without NO or LGNR were compared. Natural and synthetic rubber composites made with LGNR had similar processability to those made with NO, but had improved thermal stability, mechanical properties after aging, and ozone resistance. This was due to the strong LGNR–filler interaction and additional crosslinks formed between LGNR and the rubber matrices. The glass transition temperature of SBR composites was reduced by LGNR because of its increased molecular mobility. Thus, unlike NO, LGNR processing aid can simultaneously improve rubber composite durability, dynamic performance and renewability. The commercialization of LGNR has the potential to open a new sustainable processing-aid market.

## 1. Introduction

Rubber has been developed as an essential elastomeric material for a myriad of applications since the vulcanization process was invented in 1839 [1]. Styrene-butadiene rubber (SBR), one of the most popular synthetic rubbers, is a raw material used for tires, soles, belts and other products [2]. However, SBR is petroleum-based and non-renewable because the monomers used for SBR polymerization are products of the petroleum industry, although the bio-production of styrene is being studied [3]. Natural rubber (NR) is the only large-scale renewable rubber material and its demand is increasing because of its excellent physical properties and low-temperature flexibility [4]. However, Hevea (*Hevea brasiliensis*, Muell. Arg., commonly known as the rubber tree) natural rubber (HNR) is currently the only commercial NR. The growing area of HNR is restricted to tropical regions, mainly in Southeastern Asia, because of climatic requirements and the endemic fatal leaf blight (*Pseudocercospora ulei*) in South America [5,6]. NR production was over 11 million metric tons in 2020, which was higher than the 8 million metric tons of SBR produced in the same year [7,8]. The production of NR was over 200 thousand metric tons less than its consumption from January 2020 to November 2020 [7]. Alternative NR sources are needed to meet the increasing NR demand and to supplement HNR shortfalls. Guayule (*Parthenium argentatum*, Gray) and the rubber dandelion (*Taraxacum kok-saghyz*) are commercially promising NR plants [9]. Guayule is native to Texas and Mexico, can be farmed in the United States, and its rubber and latex does not cause latex allergies [10,11,12,13,14,15,16,17]. Compared to HNR with a branched molecular structure, guayule natural rubber (GNR) has linear molecules and more significant strain-induced crystallization, which garners interest from the rubber industry [18,19,20]. In addition, GNR is known to have a faster SIC rate than dandelion rubber [19].

Processability is important for rubber product manufacturing, since rubbers with high molecular weight are highly viscous, making it difficult to mix them uniformly with other ingredients into a rubber compound. Reinforcing filler particles can further increase viscosity, especially when they flocculate [21]. Processing oils are used to partially solubilize rubber molecules and reduce the overall mix viscosity to improve processability [22]. Processing oils also reduce the stiffness and modulus of the vulcanizate and lower the processing cost [23].

Petroleum-based mineral oils, including aromatic, paraffinic and naphthenic oil (NO), were the main processing aids in rubber products, but aromatic oil has been banned because it is a human carcinogen [24]. Safe mineral oils such as naphthenic and paraffin oils are still used as rubber plasticizers. However, these oils are non-renewable and contribute to the high carbon footprint of current rubber products. Moreover, product durability and thermal stability are compromised as mineral oils are added into rubber products [25].

Bio-based processing aids, including vegetable oils and low-molecular-weight rubber, are possible alternatives to mineral-oil-based processing aids. Palm oil, soybean oil, castor oil and cashew nut shell liquid can improve the thermal stability of natural and synthetic rubber composites when used in place of mineral oils [21,25,26,27]. The oil resistance of nitrile rubber was improved when linseed oil was used instead of dioctyl phthalate, another non-renewable commercial plasticizer [28]. Palm and soybean oils have similar effects to paraffinic oil and NO on the rheological properties of uncured ethylene–propylene–diene-monomer rubber and SBR compounds, including storage modulus, loss modulus and complex viscosity [21,26]. Cured SBR composites made with soybean oil exhibit similar aging resistance to those made with NO [22]. HNR-derived epoxidized low-molecular-weight rubber can also enhance the damping properties of carbon-black (CB)-filled NR [29].

Low-molecular-weight guayule rubber (LGNR) has a similar plasticizing effect to NO, but increases the mechanical properties when used to replace NO in natural and synthetic rubber composites [30]. However, the effect of LGNR on the durability and dynamic mechanical properties of rubber composites must be understood before LGNR can be introduced as a commercial processing aid. In this research, LGNR was used as an alternative to naphthenic oil in HNR, SBR and GNR composites. The thermal stability, dynamic mechanical properties, aging and ozone resistance of HNR, SBR and GNR composites with NO or LGNR, or without a processing aid, were characterized.

## 2. Materials and Methods

HNR (SMR L) and Emulsion SBR (Emulprene 1502) were generously provided by Momentum Technologies International (Uniontown, OH, USA). GNR was dried from guayule latex in trays at 50 °C for 120 h (HVC 70 series oven, Conceptronic Inc., Portsmouth, UK). The guayule latex was extracted from guayule plants as described [31]. LGNR was made using thermal degradation of GNR in a lab oven (OMS180, Thermo Scientific, Waltham, MA, USA) at 125 °C for 216 h. Stearic acid, zinc oxide, butyl benzothiazole sulfonamide (TBBS), antioxidant (N-(1,3-dimethylbutyl)-N’-phenyl-p-phenylenediamine (6PPD)) and sulfur were generously provided by HB chemicals (Twinsburg, OH, USA). NO (Corsol 2400, R.E. Carroll, Inc. (Ewing, NJ, USA)) was generously provided by Ford Motor Company (Dearborn, MI, USA).

Rubbers (HNR, SBR and GNR) were compounded with CB, antioxidant and curing packages (Table 1) in a Banbury mixer (Farrel-Birmingham CO, Buffalo, NY, USA) at a fill factor of 0.7. HNR compounds without fillers were mixed with antioxidant and curing packages in the same Banbury mixer at a fill factor of 0.6, to investigate the effect of LGNR and NO on unfilled rubber composites (Table 2). The weight of each CB-filled compound was constant (1292 g) to permit accurate comparison of the energy consumption of compounding, as recorded with Pro-server Ex software v1.3 (Pro-face Digital Electronics CO, Osaka, Japan). Each rubber matrix (HNR, SBR and GNR) was first mixed with 50 parts per hundred rubber (phr) CB (only for filled rubber compounds), processing aids and an antioxidant (6PPD) for 12 min; then, a curing package consisting of stearic acid, zinc oxide, TBBS and sulfur were added and mixed for an additional 3 min. After mastication, the rubber compounds were passed nine times through a two-roll mill (roll diameter 15.24 and 33.02 cm width) (Rubber City Machinery Corporation, Akron, OH, USA). Compounded samples were vulcanized using heat compression with 16 tons of force at 160 °C, according to ASTM D3182. The curing time was T_90_ plus 5 min in order to completely vulcanize each compound.

The storage modulus (G′), loss modulus (G″) and complex viscosity of CB-filled rubber compounds were measured using a Premier RPA (Alpha Technologies, Hudson, OH, USA), to characterize the effects of NO and LGNR on HNR, SBR and GNR compounds before vulcanization. Rubber compounds were tested from 0.07% to 300% strain amplitude, with a constant temperature of 90 °C and a constant frequency of 0.63 rad/s. Tan Delta (tan δ) was calculated as G″ over G′. The G′s of HNR compounds made with 10 phr NO or LGNR, but no filler, were measured using an RPA 2000 (Alpha Technologies, Hudson, OH, USA) similar to Premier RPA, before vulcanization. Rubber compounds were tested from 0.5% to 1% strain amplitude, with constant temperature and frequency of 70 °C and 6.3 rad/s, respectively. The mean Gs at various strain amplitudes were statistically compared using *t*-tests (α = 0.05) in JMP Pro 12 software (SAS Institute Inc., Cary, NC, USA).

Thermal stability was analyzed using a thermogravimetric analyzer (TGA Q50, TA instruments, New Castle, DE, USA). Cured rubber composites were heated from 25 °C to 800 °C with a heating rate of 20 °C/min. The temperature at which samples lost 5% of their weight was recorded to evaluate the thermal stability.

The heat–flow curves and glass transition temperature of composites made with NO and LGNR and without processing aids were measured using differential scanning calorimetry (DSC 2500, TA instruments, New Castle, DE, USA) under a nitrogen atmosphere. The samples were first heated to 80 °C at a rate of 10 °C/min and kept at 80 °C for 5 min, in order to eliminate thermal history. Then, the samples were cooled to −90 °C at a cooling rate of 10 °C/min before starting the tests. Finally, the heat flow was recorded as samples were heated up from −90 to 50 °C at a heating rate of 10 °C/min. Glass transition temperatures were analyzed with the TA Universal Analysis software (TA instruments, New Castle, DE, USA).

The loss modulus E″ and storage modulus E′ of the cured composites were measured using a dynamic mechanical analyzer Q800 (TA Instruments, New Castle, DE, USA), using a sample size of 18 mm × 3 mm × 2 mm (length × width × thickness). Testing conditions were 1 Hz from −90 °C to 90 °C at 3°C/min. The 0.5% strain amplitude, tan δ, which estimates the relative amount of viscous and elastic portions in the composite, was calculated using the ratio of E″ over E′. Rolling resistance (energy loss during continuous deformation) and wet grip (friction between wet surface and rubber material) were estimated using tan δ at 60 °C and 0 °C, respectively [32,33,34].

The evaluation of static and dynamic stiffness is particularly important to understand the performance of the materials used in anti-vibration applications. Natural rubber is the most common polymer used in anti-vibration applications due to its unique viscoelastic behavior and strain-induced crystallization, so only GNR and HNR were evaluated for this purpose.

The static stiffness (Ks) was determined according to ASTM D575. Rubber discs 12.8 mm tall and 28.6 mm in diameter were tested under compression at a rate of 12 ± 3 mm/min using an MTS Model 831 (Eden Prairie, MN, USA). In order to remove Mullin’s effect, mechanical conditioning of the samples was performed by applying two pre-cycles followed by the measurement of force vs. displacement in the third cycle. The slope of the linear region of the force vs. displacement curve was reported as the static stiffness.

There are currently no defined standard testing conditions for dynamic stiffness. Hence, the testing conditions used in this study were selected based on the existing literature and considering general guidelines for the dynamic testing of elastomers using vibratory methods (ASTM D5992-96). The dynamic properties of elastomers are dependent on the frequency, amplitude and preload applied to the polymer. For the screening and comparison of new materials, frequency sweeps at multiple amplitudes are recommended. In this study, dynamic stiffness (Kd), phase angle and transmissibility (Tr) were evaluated as a function of frequency (1–500 Hz). Amplitude selection depends on the frequency of the vibration. For instance, low frequencies (0–150 Hz) are mainly associated with high-amplitude vibrations, and high frequency (>150 Hz) occurs at low amplitudes. The preload used in the dynamic testing is based on the load deflection behavior of the material. Therefore, two different amplitudes (0.01 and 0.316 mm peak-to-peak (p-p)) were tested using a 500 N pre-load. The same cylindrical sample shape used for the static testing was used for the dynamic testing. All samples were evaluated at room temperature using an MTS Model 831.

Cured rubber composites were cut with a die D (CCSi Inc., Akron, OH, USA) to prepare accelerated aging samples, according to ASTM D412. Accelerated aging samples were suspended vertically in a lab oven (Quincy Lab Inc., Chicago, IL, USA) for 168 h at 100 °C, according to ASTM D 573. After aging, the mechanical properties, namely tensile strength, modulus at 100% and elongation at break, were measured using a tensiometer (Model 3366, Instron, Norwood, MA, USA) at ambient temperature (23 ± 2 °C), according to ASTM D412. The strain level was calibrated using an elongation axial extensometer (Model 3800, Epsilon Technology Corp., Jackson, WY, USA). The hardness number of the aged samples was measured using a Shore A durometer (Model 408, PTC Instruments, Los Angeles, CA, USA) fixed on an operating stand type 2 (Model 472, PTC Instruments, Los Angeles, CA, USA), according to ASTM D 2240.

The accelerated aging samples (0.5 g) were immersed in toluene to measure the crosslink density, according to the Flory–Rehner equation [35]:

(1) $$-\ln\left(1-V_{r}\right)-V_{r}-\chi V_{r}{}^{2}=V_{s}\eta_{\mathrm{swell}}\left(V_{r}^{\frac{1}{3}}-\frac{V_{r}}{2}\right)$$

where χ is the rubber–solvent interaction parameter; χ for HNR-toluene, SBR-toluene and GNR-toluene are 0.391, 0.310 and 0.391, respectively [36,37]; $\eta_{\mathrm{swell}}$ is the crosslink density of rubber (kmol/m^3^); and the molar volume of toluene, $V_{s}$, is 106.27 cm^3^/mol [38]. The volume fraction of the rubber in swollen gel, *V_r_*, was measured by the equation:

(2) $$V_{r}=\frac{V_{rubber}}{V_{solvent}+V_{rubber}}=\left(\frac{m_{d}-m_{b}\times f}{\rho_{rubber}}\right)÷\left[\frac{m_{s}-m_{d}}{\rho_{solvent}}+\left(\frac{m_{d}-m_{b}\times f}{\rho_{rubber}}\right)\right]$$

where $V_{rubber}$ and $V_{solvent}$ are the volume of rubber matrix and toluene in swollen gel, which are calculated by weight and density; $m_{b},m_{s},\;m_{d}$ are the weights of the sample: $m_{b}$ is the sample weight before swelling tests, $m_{s}$ is swollen weight and $m_{d}$ is the weight measured after drying the swollen samples; $\rho_{rubber}$ and $\rho_{solvent}$ are the densities of the rubber matrices (HNR, SBR and GNR) and toluene, respectively; $\rho_{rubber}$ of HNR, SBR and GNR were 0.91, 0.85 and 0.92 g/cm^3^, which were measured using an analytical balance (Model ME54E, Mettler Toledo, Columbus, OH, USA); $\rho_{solvent}$ was 0.867 g/cm^3^; and f is the weight fraction of the non-rubber components (filler, curing packages, processing aids and antioxidant).

$m_{b},m_{s},\;m_{d}$ of the cured rubber samples were weighed to an accuracy of 1 mg using an analytical balance (Model ME54E, Mettler Toledo, Columbus, OH, USA). Samples were immersed in 40 mL toluene at 21 °C for 96 h. Toluene was replaced every 24 h, according to ASTM D6814. The $m_{s}$ of the swelled sample was weighed after samples were blotted with clean wiper, then $m_{d}$ was weighed after 24 h of drying the swollen samples at 100 °C.

As described in ASTM D 1149, the HNR, SBR and GNR composites were cut into rectangular strips of 100 mm × 10 mm × 21 mm (length × width × thickness). The strips were clamped in an environmental test chamber (Corporate Consulting Services Inc, Akron OH) for 10 h, at 40 °C, in an ozone concentration of 0.001 mg/L. During the treatment, samples were stretched and released repeatedly at 0.5 Hz, within a 0–25% strain. After ozone treatment, the crack ratio of the stretched samples at a 20% strain was calculated using optical microphotographs at a 6.3× magnification (Leica Camera, Wetzlar, Germany). The crack ratio was calculated as the ratio of the crack area to the total area of the sample surfaces. A higher crack ratio indicates worse ozone resistance, so ozone resistance was calculated as the reciprocal of the crack ratio.

## 3. Results and Discussion

### 3.1. Rheology Tests

**Figure 1:**
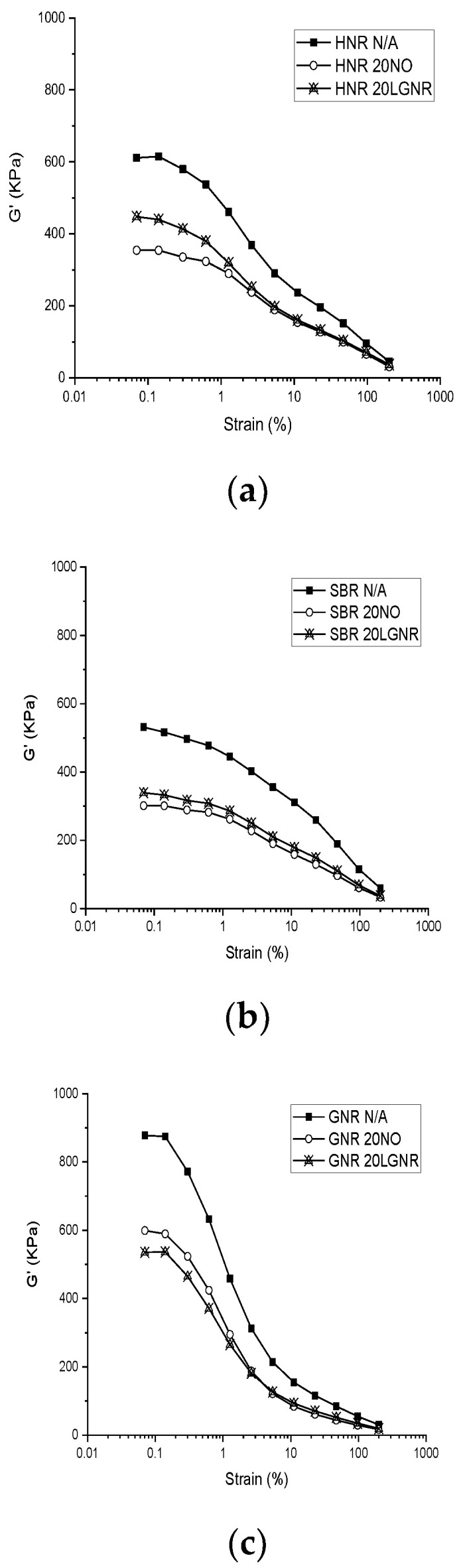
Storage modulus (G′) of rubber composites made with or without processing aids from 0.07% to 200% strain amplitude: (**a**) Hevea natural rubber (HNR); (**b**) styrene butadiene rubber (SBR); (**c**) guayule natural rubber (GNR). N/A: rubber compounds without processing aids; 20 NO: rubber compounds with 20 phr naphthenic oil; 20 LGNR: rubber compounds with 20 phr liquid guayule natural rubber.

**Figure 2:**
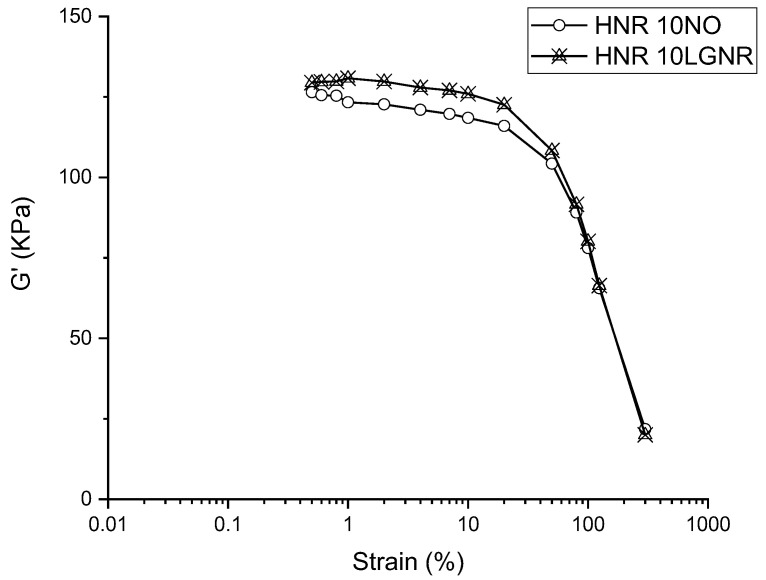
Storage modulus (G′) of unfilled Hevea natural rubber (HNR) compounds with LGNR or NO from 0.5% to 300% strain amplitude. 10NO: rubber compounds with 10 phr naphthenic oil; 10LGNR: rubber compounds with 10 phr liquid guayule natural rubber.

The values of the G′ of all rubber compounds decreased with increasing strain because of the “Payne effect” [32] (Figure 1 and Figure 2), caused by filler–filler interactions at low strain levels. CB networks formed in the rubber compound can trap rubber molecules and restrict the mobility of rubber, resulting in a higher G′ and an effective filler volume fraction at a low strain level. However, the rubber releases after the CB networks are broken at a high strain level. Therefore, the modulus and effective filler volume fraction are reduced at a high strain amplitude. Both LGNR and NO had lowered G′ compared to the rubber compounds without processing aids, which can be explained by the plasticizing effects of LGNR and NO.

**Figure 3:**
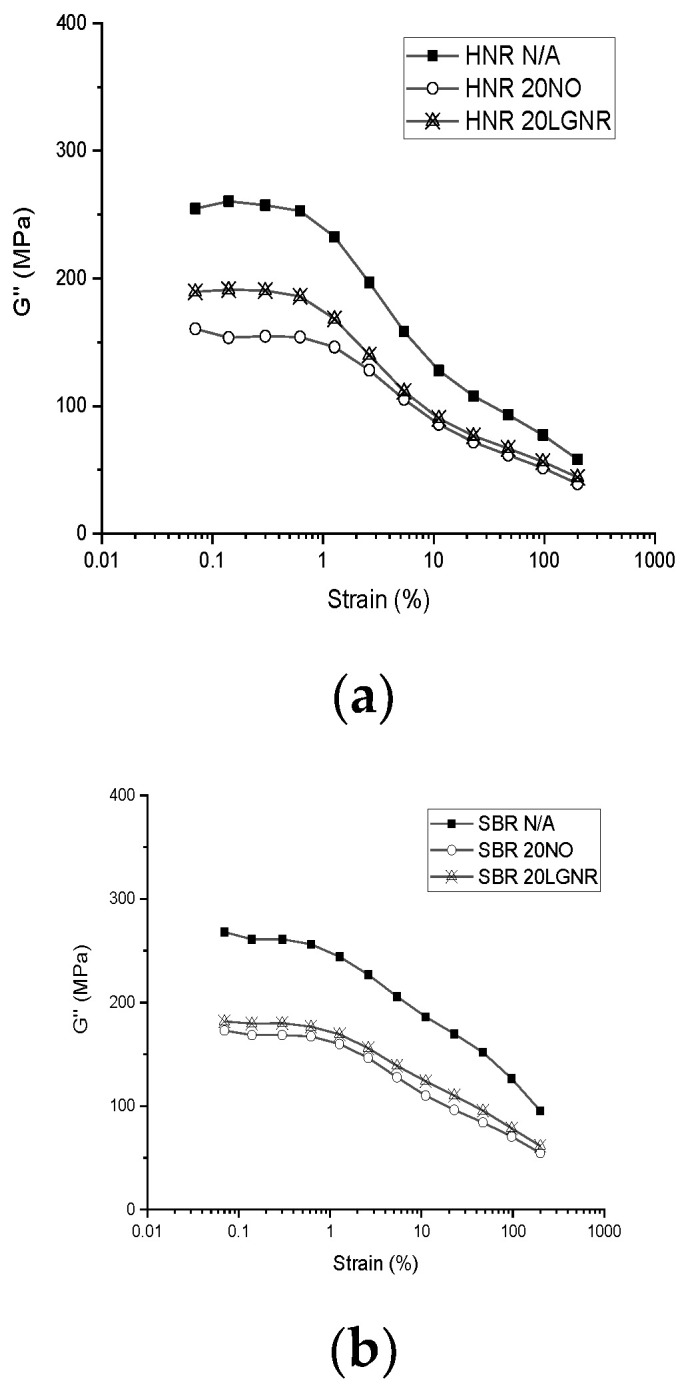

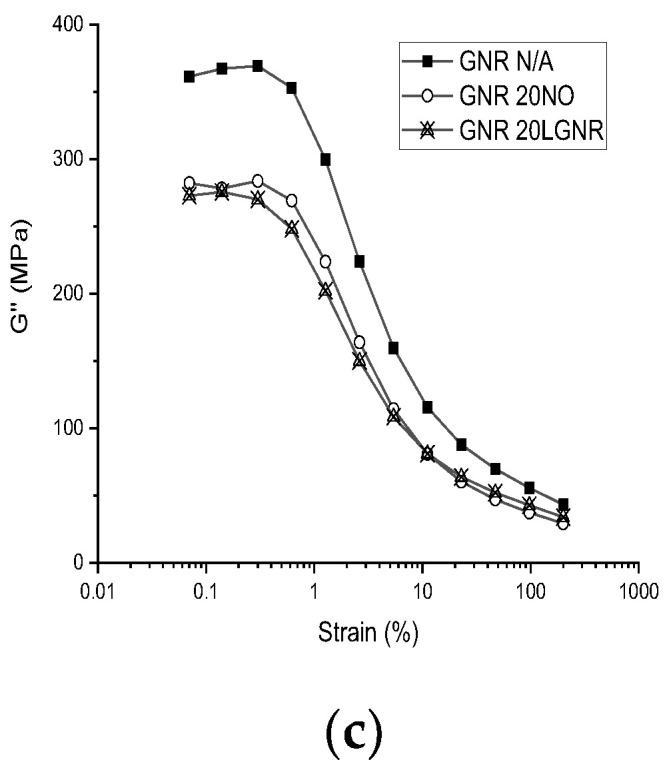
Loss modulus (G″) of rubber compounds with or without processing aids from 0.07% to 200% strain amplitude: (**a**) Hevea natural rubber (HNR); (**b**) styrene butadiene rubber (SBR); (**c**) guayule natural rubber (GNR). N/A: rubber compounds without processing aids; 20 NO: rubber compounds with 20 phr naphthenic oil; 20 LGNR: rubber compounds with 20 phr liquid guayule natural rubber.

HNR and SBR compounds made with LGNR had higher G′ than the ones made with NO (Figure 1 and Figure 2), which can be explained by strong interactions between the LGNR and rubber matrices [39]. The molecular weight of LGNR was 80,570 g/mol, substantially higher than the 540 g/mol of NO. The long-chain structures of LGNR increased effective intermolecular friction and rubber chain entanglements. In addition, strong LGNR–rubber and LGNR–CB interactions enhanced the hysteresis and effective volume fraction of fillers, which resulted in higher G″ than those made with NO [32](Figure 3).

**Figure 4:**
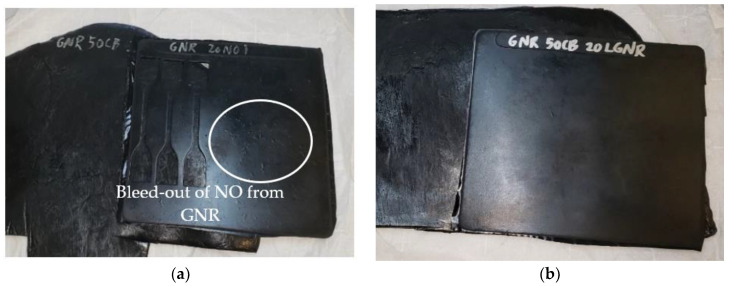
Cured guayule natural rubber (GNR) composites: (**a**) with 10 phr naphthenic oil; and (**b**) with 10 phr liquid guayule natural rubber.

Unlike HNR and SBR compounds, GNR compounds made with LGNR had lower G′ and G″ than those made with NO, because NO has poor compatibility with GNR, and the plasticizing effect of NO in GNR compounds is limited. (Figure 1). LGNR derived from GNR has some structural similarities and, so, has good compatibility; this is evinced by the homogenous and smooth appearance of the composite (Figure 4b). In contrast, NO was poorly compatible with GNR, and NO caused blistering of the composite surface as NO bled form the composite (Figure 4a).

**Figure 5:**
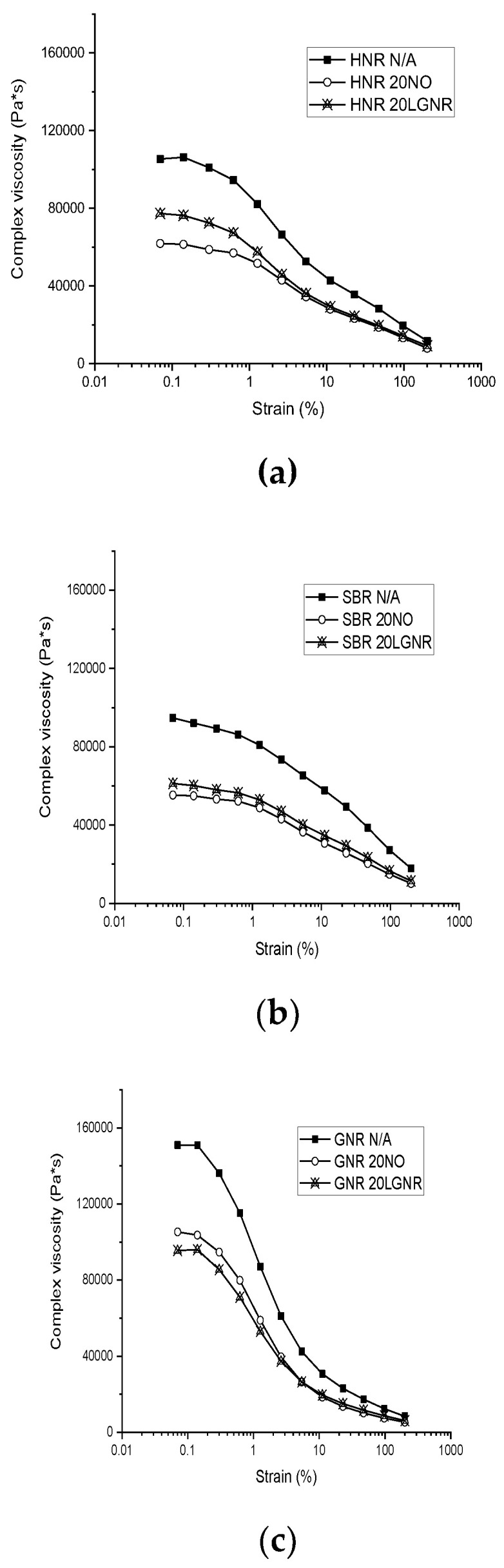
Complex viscosity of rubber compounds with or without processing aids from 0.07% to 200% strain amplitude: (**a**) *Hevea* natural rubber (HNR); (**b**) styrene butadiene rubber (SBR); (**c**) guayule natural rubber (GNR). N/A: rubber compounds without processing aids; 20 NO: rubber compounds with 20 phr naphthenic oil; 20 LGNR: rubber compounds with 20 phr liquid guayule natural rubber.

Both processing aids reduced the complex viscosity, which indicates that LGNR can facilitate rubber processing by reducing compound viscosity (Figure 5). The complex viscosity of HNR and SBR compounds made with LGNR was slightly higher than those made with NO, due to the high molecular weight of LGNR. In contrast, GNR compounds with LGNR had lower complex viscosity than GNR compounds with NO due to the poor compatibility of NO with GNR.

### 3.2. Thermal Analysis

**Figure 6:**
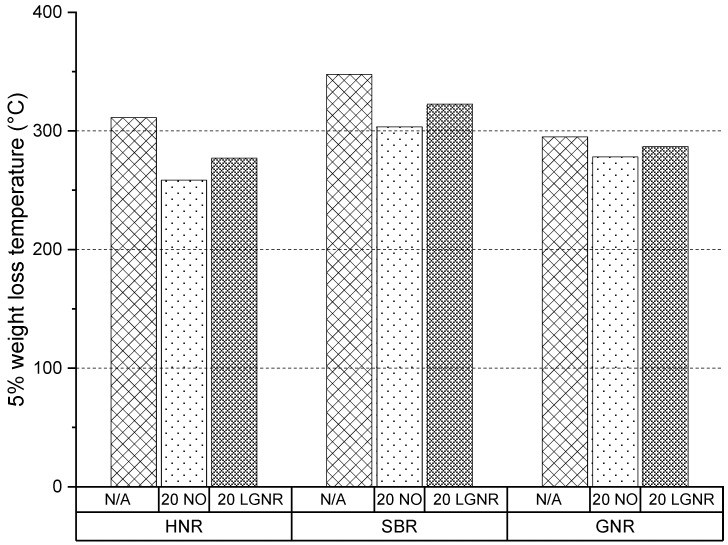
The 5% weight-loss temperature of HNR, SBR and GNR composites made with or without processing aids at 5% weight loss. N/A: rubber compounds without processing aids; 20 NO: rubber compounds with 20 phr naphthenic oil; 20 LGNR: rubber compounds with 20 phr liquid guayule natural rubber.

**Figure 7:**
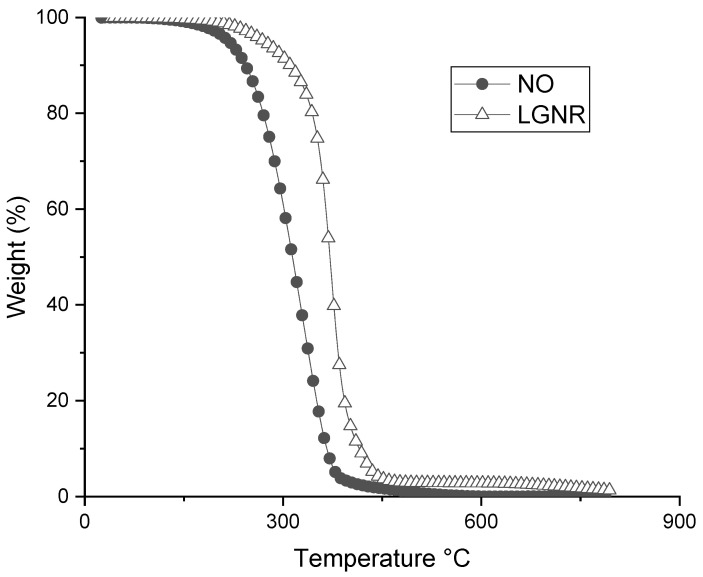
Weight–temperature curves of NO and LGNR. NO: naphthenic oil; LGNR: liquid guayule natural rubber.

SBR composites had higher thermal stability than HNR and GNR composites (Figure 6). All the rubber composites made with LGNR had higher thermal stability than those with NO, but lower stability than the rubber composites made without processing aids (Figure 6). The improved thermal stability was due to the long-chain structure of LGNR. The molecular weight of LGNR was higher than NO, but lower than the rubber matrices [30]. Thus, thermal stability was improved by replacing NO with LGNR (Figure 7).

**Figure 8:**
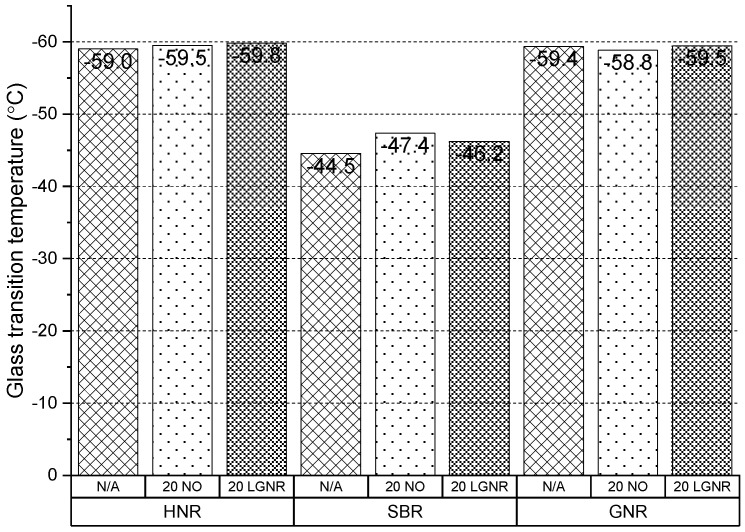
Glass transition temperatures of HNR, SBR and GNR composites made with or without processing aids. N/A: rubber compounds without processing aids; 20 NO: rubber compounds with 20 phr naphthenic oil; 20 LGNR: rubber compounds with 20 phr liquid guayule natural rubber.

LGNR and NO had little effect on the glass transition temperature of HNR and GNR rubber composites but reduced the glass transition temperature of SBR composites (Figure 8). The different glass transition temperatures of the rubber matrices and processing aids may explain this phenomenon. The glass transition temperatures of the GNR and HNR composites without the processing aids were −59.4 and −59.0 °C, respectively, which were close to the glass transition temperatures of NO and LGNR (Table 3). However, the glass transition temperature of the SBR composite was −44.5 °C, higher than NO and LGNR.

### 3.3. Dynamic Mechanical Analysis

**Figure 9:**
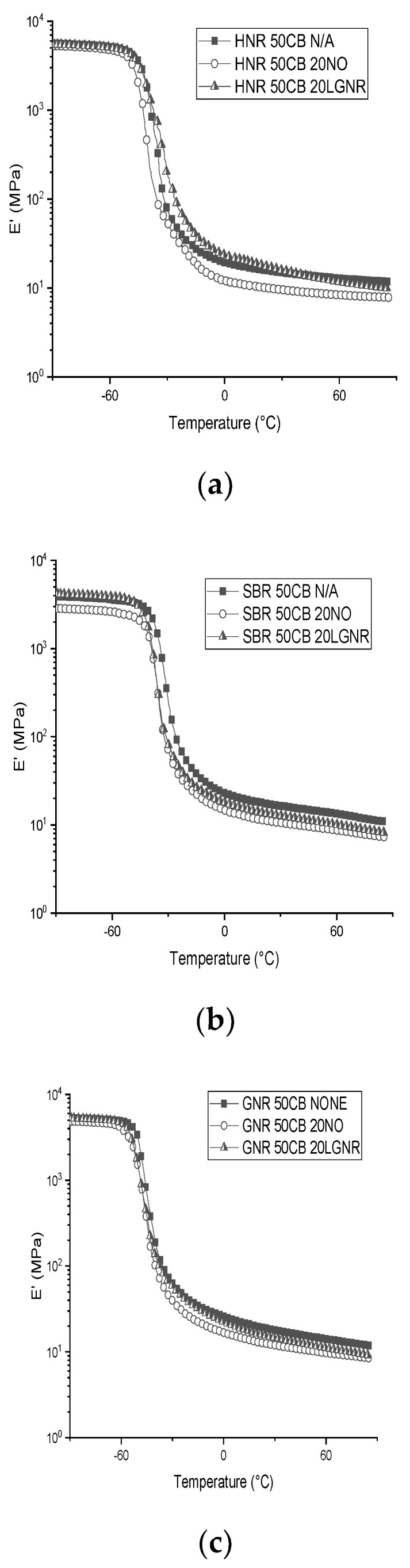
Storage modulus (E′) of rubber composites made with or without processing aids from −90 °C to 90 °C: (**a**) Hevea natural rubber (HNR); (**b**) styrene butadiene rubber (SBR); (**c**) guayule natural rubber (GNR). N/A: rubber compounds without processing aids; 20 NO: rubber compounds with 20 phr naphthenic oil; 20 LGNR: rubber compounds with 20 phr liquid guayule natural rubber.

The E′ of GNR and HNR composites was reduced by NO, but changed little with LGNR (Figure 9a,c). SBR composites made with LGNR had higher E′ than those made with NO, and SBR composites made without processing aids had the highest E′ (Figure 9b). The additional crosslinks formed between LGNR and rubber may explain the higher E′ of rubber composites made with LGNR, compared to those made with NO. In addition, the reinforcing effect of LGNR discussed in our previous study (Ren et al., 2020) can explain the higher E′ of rubber composites made with LGNR than with NO, because LGNR increases rubber–filler interactions and the effective filler volume fraction [21].

**Figure 10:**
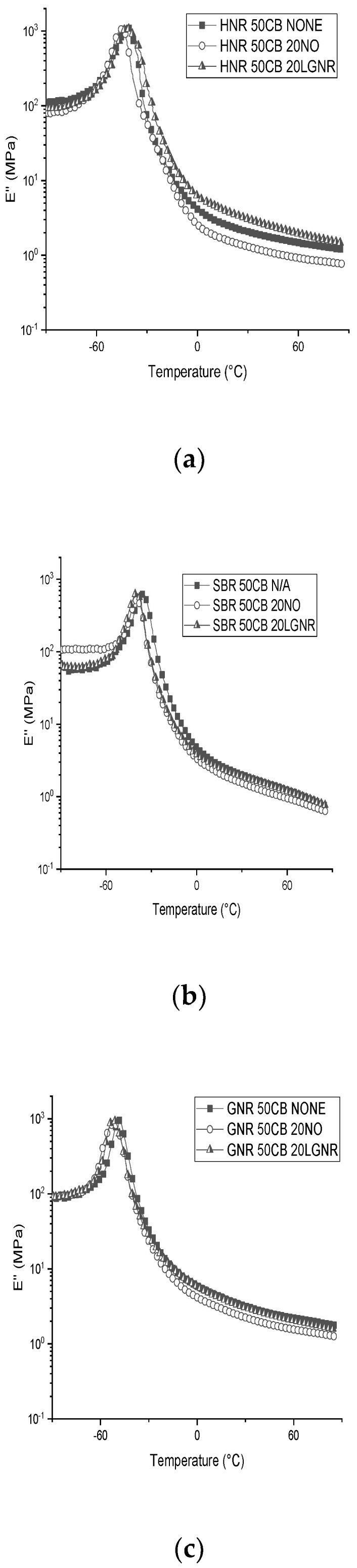
Loss modulus (E″) of rubber composites made with or without processing aids from −90 °C to 90 °C: (**a**) Hevea natural rubber (HNR); (**b**) styrene butadiene rubber (SBR); (**c**) guayule natural rubber (GNR). N/A: rubber compounds without processing aids; 20 NO: rubber compounds with 20 phr naphthenic oil; 20 LGNR: rubber compounds with 20 phr liquid guayule natural rubber.

The E”s of HNR and GNR composites were independent of the added processing aids in their glass states, because the rubber matrices and processing aids had similar glass transition temperatures (Figure 8 and Figure 10a,c and Table 3). However, SBR composites made with NO had higher E”s than the other two SBR composites, due to the high plasticizing effect of NO (Figure 10b). Although LGNR also acted as a processing aid, the additional crosslinks formed between LGNR and rubber reduced the mobility of SBR molecules. E” peaks indicate the glass transition zone of rubber composites where rubber composites transfer from glass to a rubbery state with increasing temperature. In the rubbery state, the E”s of LGNR rubber composites were higher than those of NO, but were similar to the rubber composites without processing aids; this is because the long-chain structures of LGNR increased the intermolecular friction and energy dissipation under dynamic loadings. The molecular weight of LGNR is about 80,000 g/mol—much larger than that of NO (540 g/mol). In contrast, low-molecular-weight NO reduced the viscoelasticity of the rubber composites, similar to what was observed when jet fuel was added to nitrile rubber composites [40].

**Figure 11:**
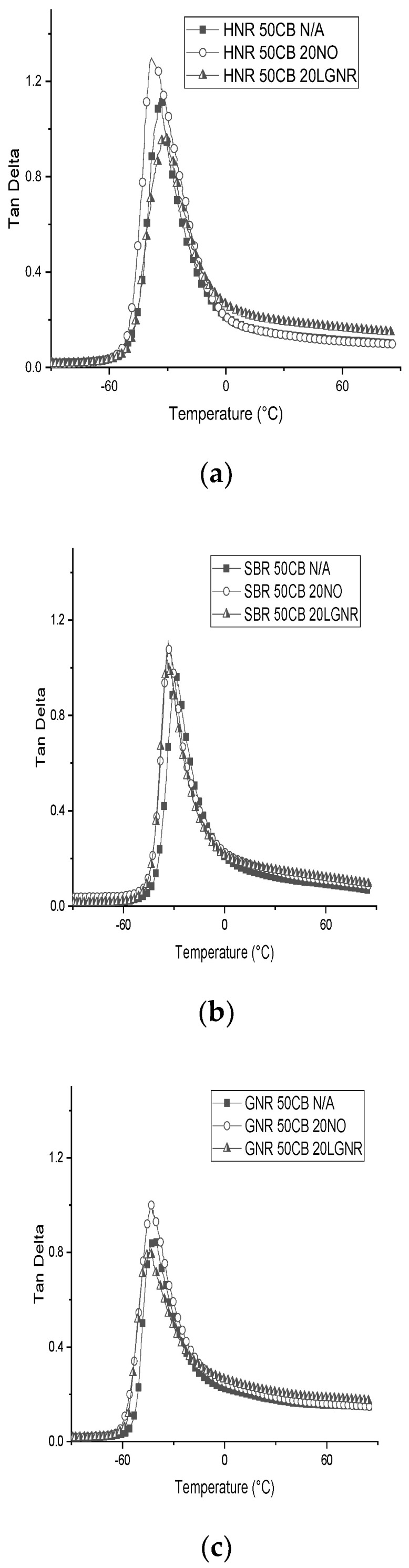
The tan δ of rubber composites made with or without processing aids from −90 °C to 90 °C: (**a**) Hevea natural rubber (HNR); (**b**) styrene butadiene rubber (SBR); (**c**) guayule natural rubber (GNR) composites. N/A: rubber compounds without processing aids; 20 NO: rubber compounds with 20 phr naphthenic oil; 20 LGNR: rubber compounds with 20 phr liquid guayule natural rubber.

Rubber composites made with NO had higher tan δ peaks than those with or without LGNR because of the plasticizing effect of NO (Figure 11a–c). Low-molecular-weight NO can increase the free space between rubber molecules and the mobility of rubber molecules. In contrast, the additional crosslinks formed by LGNR and strong LGNR–CB interactions may restrict the mobility of rubber molecules, resulting in lower tan δ peaks of rubber composites made with LGNR. These tan δ peaks were also lower for rubber composites made without processing aids. The tan δ peaks of NO and LGNR SBR composites were further to the left than the tan δ peaks of SBR composites made without processing aids (Figure 11b), because both processing aids lowered the glass transition temperature of the SBR composites.

The tan δ at 0 and 60 °C is generally used to estimate wet traction and rolling resistance, respectively, for tire applications [32]. Both LGNR and NO had little effect on the estimated wet traction (tan δ at 0 °C) of any of the rubber composites (Table 4). The estimated rolling resistance (tan δ at 60 °C) was slightly increased by LGNR, but was similar as composites with or without NO.

### 3.4. Static and Dynamic Stiffness

**Figure 12:**
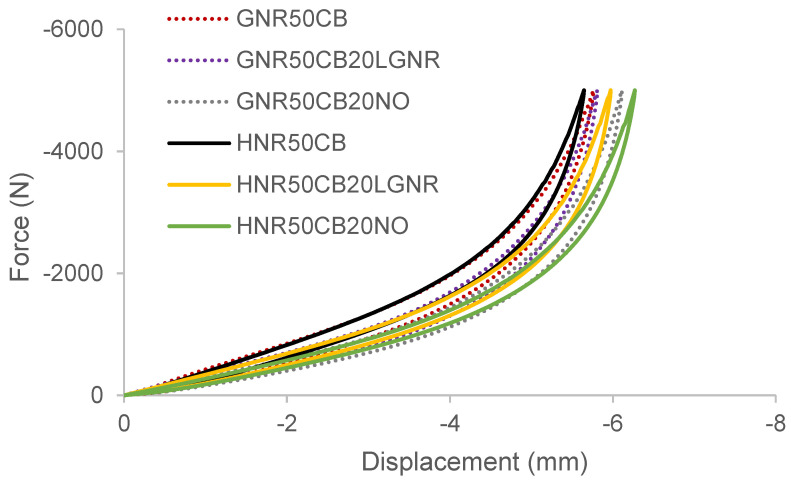
Force–displacement curves of natural rubber composites.

All the GNR and HNR composites were able to withstand the same maximum load in compression (5kN). However, composites containing processing aids had a lower Ks to those without processing aids, as shown by the larger displacements at equal load (Figure 12). This is due to the softening effect of lower-molecular-weight processing aids. Nevertheless, similar to with the E′s, LGNR increased the Ks of the composites when used instead of NO.

**Figure 13:**
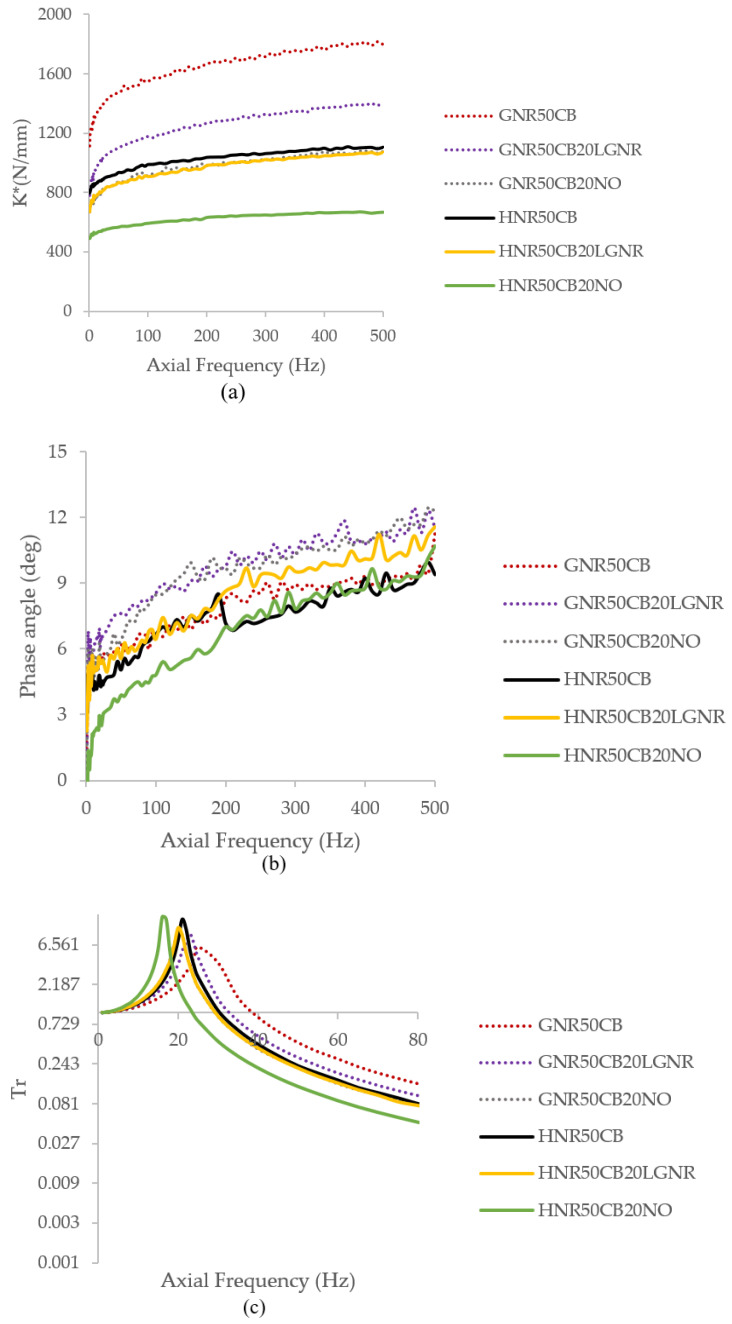
(**a**) Dynamic stiffness represented by the complex stiffness (K*); (**b**) phase angle; and (**c**) transmissibility of natural rubber composites at 0.01mm peak-to-peak amplitude.

**Figure 14:**
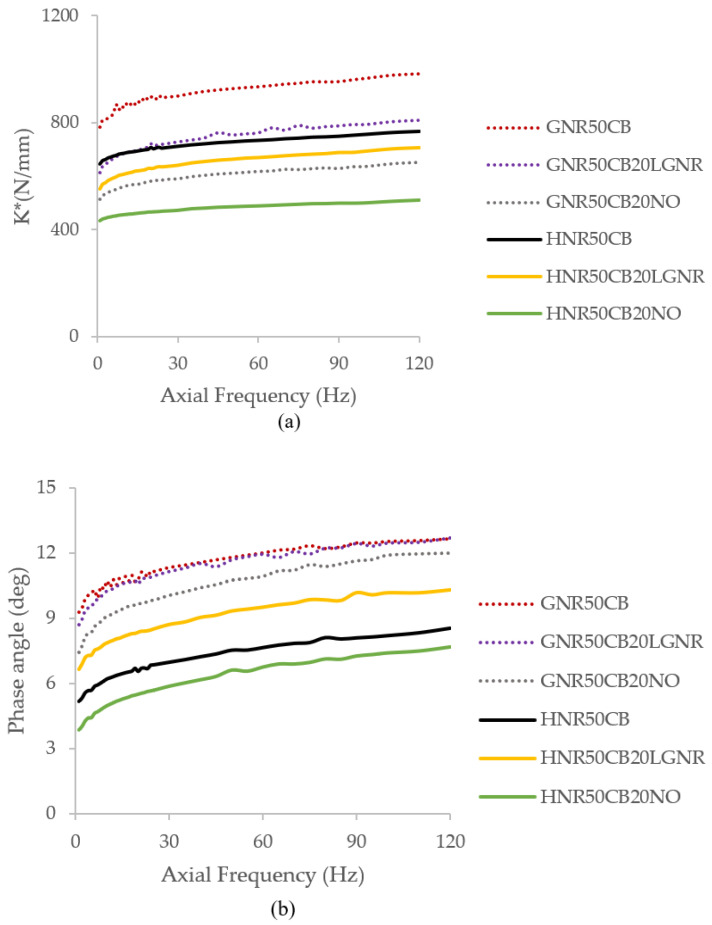

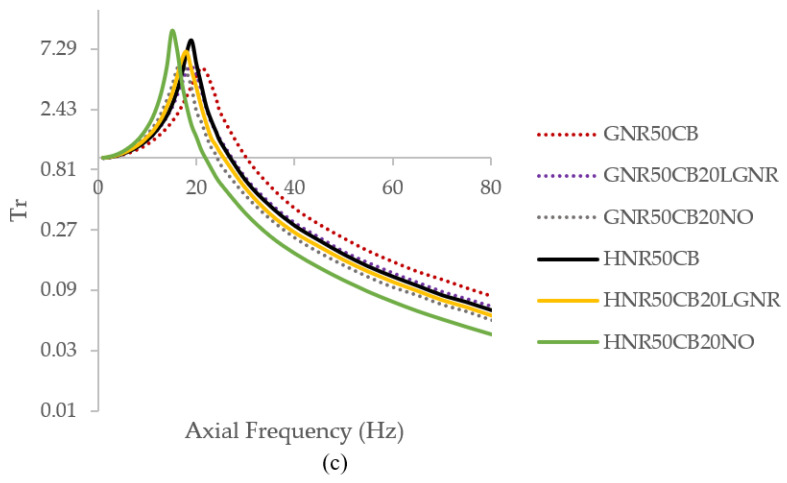
(**a**) Dynamic stiffness represented by the complex stiffness (K*); (**b**) phase angle; and (**c**) transmissibility of natural rubber composites at, 0.316 mm peak-to-peak amplitude.

The dynamic stiffness (Kd) of GNR, represented by the complex stiffness K* parameter, was reduced by both processing aids at both amplitudes evaluated (Figure 13a and Figure 14a). Nevertheless, GNR composites containing LGNR had higher Kd than composites made with NO. A similar trend was obtained for HNR composites, although the Kd of the HNR composite with LGNR was closer to that of the HNR composites without processing aids. The higher Ks and Kd of LGNR composites can be explained by a higher crosslink density and a good polymer–filler interaction, which increased the effective filler volume.

The damping of elastomers represents the ability of the material to transform applied mechanical energy to heat. Therefore, it is the result of internal friction caused by the rearrangement of polymers. By increasing the free volume with the addition of a plasticizer, the polymer chains can better rearrange and dissipate the energy. HNR made with LGNR had similar damping behavior to composites without processing aids at 0.01mm, and more damping than those containing NO (Figure 13b). Meanwhile, GNR composites made with processing aids had more damping than those without. At the higher amplitude of 0.316 mm (Figure 14b), GNR composites containing LGNR had similar damping to composites made without processing aids, and more than those made with NO, while HNR made with LGNR had the most damping among the HNR composites. NO does not add to the hysteresis of the material, but LGNR can add both a plasticizing effect and more damping to both rubbers. This is due to the presence of lower-molecular-weight polymer and pendant chains, which increase hysteresis as a result of chain slipping when the strain is applied.

Isolation and damping are different but related properties. Isolation of a material is determined by measuring the force transmitted through the material. At the peak of the transmissibility (Tr) curve, also known as the natural frequency of the material, there is amplification of the vibration. The extent of the peak amplitude depends on the amount of damping provided by the material. Beyond the natural frequency, most of the vibrations are suppressed. The addition of processing aids shifted the natural frequency of the composites to a lower frequency, while also narrowing the range of frequencies transmitted (Figure 13c and Figure 14c). However, this shift was smaller for HNR and GNR composites made with LGNR, which had transmissibility curves very similar to the composites without processing aids. This behavior is consistent with the decreased dynamic stiffness obtained. The natural frequency of a material is directly proportional to the stiffness and inversely proportional to the mass of the sample. Therefore, the overall results indicate that the addition of LGNR can have an additive function, to enhance the material’s performance for damping applications in addition to serving as processing aid.

### 3.5. Aged Mechanical Properties

**Figure 15:**
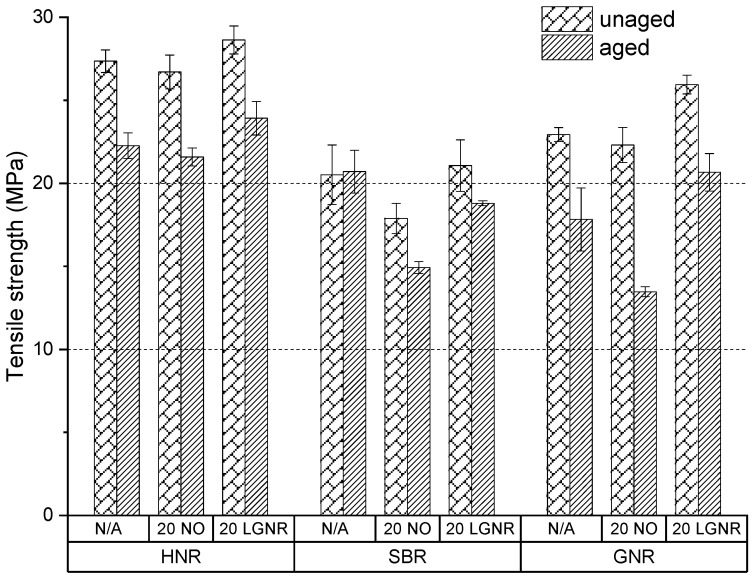
Aging effects on the tensile strength of HNR, SBR and GNR composites made with or without processing aids. N/A: rubber compounds without processing aids; 20 NO: rubber compounds with 20 phr naphthenic oil; 20 LGNR: rubber compounds with 20 phr liquid guayule natural rubber.

**Figure 16:**
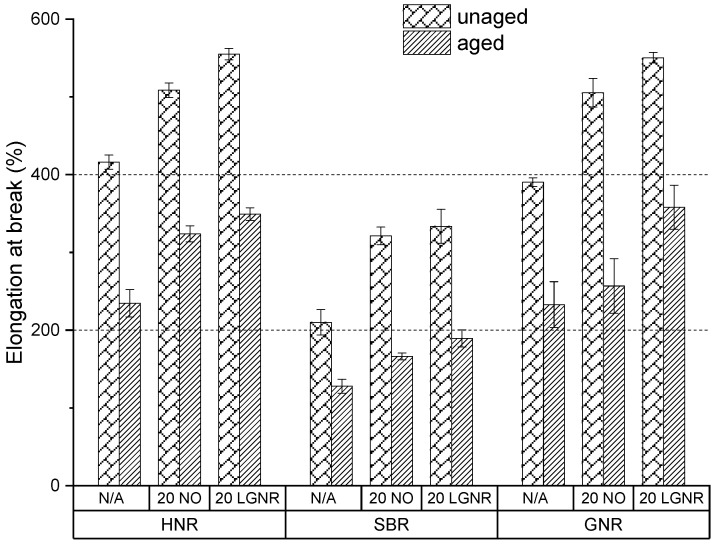
Aging effects on the elongation at break of HNR, SBR and GNR composites made with or without processing aids. N/A: rubber compounds without processing aids; 20 NO: rubber compounds with 20 phr naphthenic oil; 20 LGNR: rubber compounds with 20 phr liquid guayule natural rubber.

**Figure 17:**
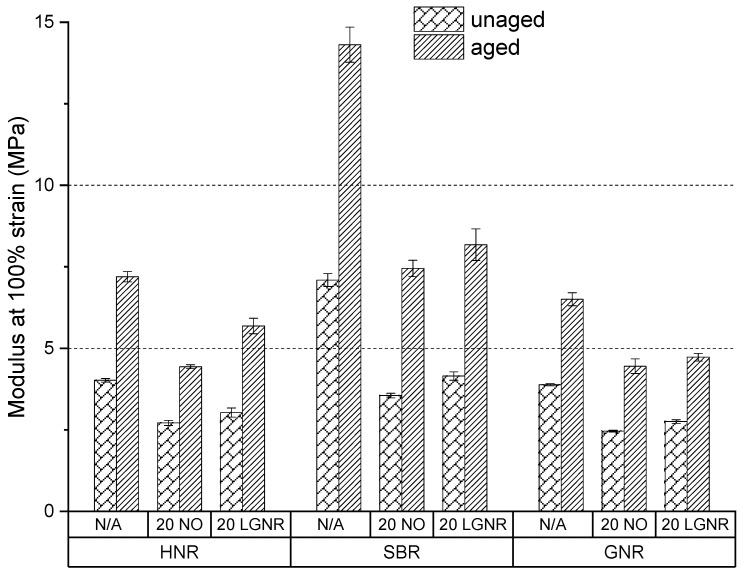
Aging effects on the modulus at 100% strain (M100) of HNR, SBR and GNR composites made with or without processing aids. N/A: rubber compounds without processing aids; 20 NO: rubber compounds with 20 phr naphthenic oil; 20 LGNR: rubber compounds with 20 phr liquid guayule natural rubber.

**Figure 18:**
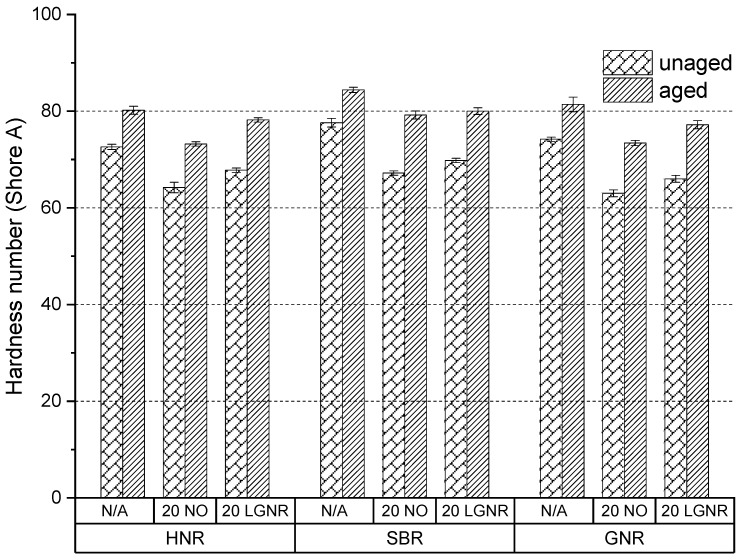
Aging effects on the hardness number of HNR, SBR and GNR composites made with or without processing aids. N/A: rubber compounds without processing aids; 20 NO: rubber compounds with 20 phr naphthenic oil; 20 LGNR: rubber compounds with 20 phr liquid guayule natural rubber.

Aging reduced the tensile strength and elongation at break but increased the modulus at 100% strain and the hardness number, because of post-curing and oxidative coupling (Figure 15, Figure 16, Figure 17 and Figure 18). Free sulfur remaining in the cured composites with conventional curing system continues to crosslink rubber molecules during aging in all natural and synthetic rubber composites [41,42,43]. Oxidative groups formed during aging, such as -C=O and -C-OH, can react to form oxidation-induced crosslinking structures, such as -C-O-O-C-, which also increase crosslink density and stiffness; however, they reduce elongation at break, as well as tensile strength [44,45].

LGNR increased the tensile strength, elongation at break, modulus at 100% strain and hardness number of natural and synthetic rubber composites when used to replace NO, as was discussed in our previous study [30]. Aged HNR, SBR and GNR composites made with LGNR also had higher tensile strength, elongation at break, modulus at 100% strain and hardness number than composites made with NO, which is attributed to strong LGNR–CB interactions.

LGNR may act as an anti-aging agent in rubber composites by diverting chain scission to the unsaturated bonds of LGNR, reducing scission of the rubbers’ backbones. This was not seen in NO composites because NO only acted as a plasticizer and had no reaction with oxygen.

**Figure 19:**
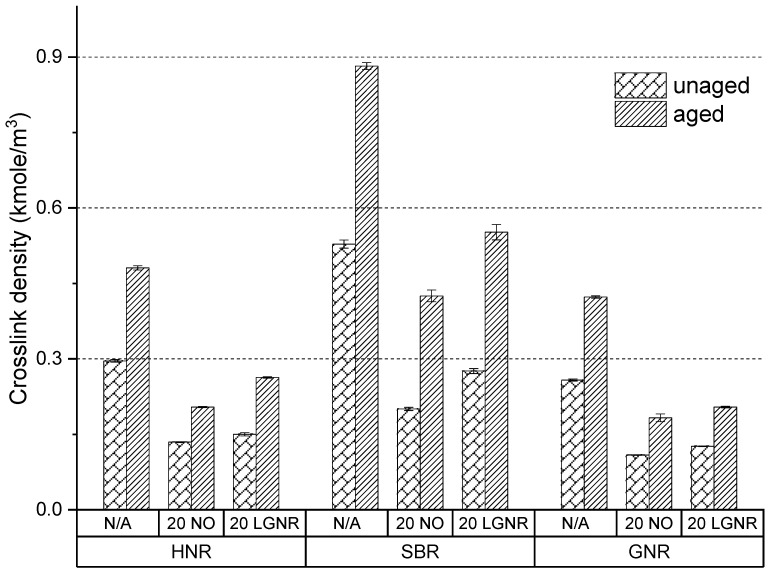
Aging effects on the crosslink density of HNR, SBR and GNR composites made with or without processing aids. N/A: rubber compounds without processing aids; 20 NO: rubber compounds with 20 phr naphthenic oil; 20 LGNR: rubber compounds with 20 phr liquid guayule natural rubber.

Accelerated aging increased the crosslink density of all rubber composites, confirming that post-curing and oxidative coupling occurred (Figure 19). SBR composites had higher-aged crosslink density than HNR and GNR composites. All rubber composites made with LGNR had higher-aged crosslink density than those made with NO, but lower-aged ones than those made without processing aids. Additional LGNR–LGNR and LGNR–rubber crosslinks can be formed by post-curing due to the existence of unsaturated bonds in LGNR. NO lacks unsaturated bonds and, so, only acts as a plasticizer by increasing the distance between rubber molecules.

### 3.6. Ozone Resistance

**Figure 20:**
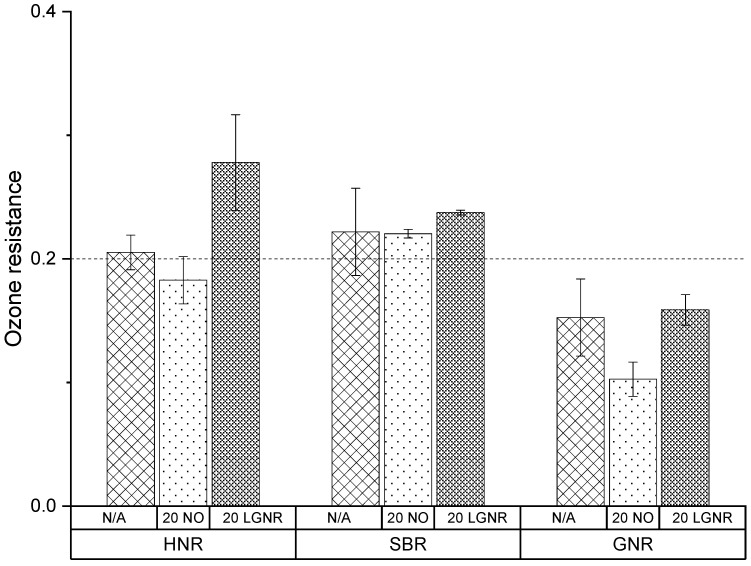
Ozone resistance of HNR, SBR and GNR composites made with or without processing aids. N/A: rubber compounds without processing aids; 20 NO: rubber compounds with 20 phr naphthenic oil; 20 LGNR: rubber compounds with 20 phr liquid guayule natural rubber.

GNR composites were more sensitive to ozone than HNR and SBR composites (Figure 20). The processing aids had little effect on the ozone resistance of SBR composites, but NO reduced the ozone resistance of both HNR and GNR composites. Adding LGNR as a processing aid to rubber composites maintained the ozone resistance of the SBR and GNR composites and increased the ozone resistance of HNR composites. LGNR produced by thermal degradation contains oxidative groups such as -C=O and -C-O-C-, and those oxidative groups are more stable than the unsaturated bonds in ozone. In addition, unsaturated bonds in LGNR can also scavenge free radicals generated by ozone. Thus, LGNR can effectively protect rubber composites from an ozone attack.

## 4. Conclusions

LGNR—an innovative, sustainable, biobased processing aid—not only behaves as a processing aid but improves the properties of cured rubber composites; this is because, unlike diluent conventional aids, it is an active participant in the vulcanization reaction. LGNR improved the processability, thermal stability and ozone resistance of natural and synthetic rubber composites when used to replace petroleum-based NO. Unlike the opposite changes generally observed in NO, both flexibility and stiffness increased with LGNR. Natural and synthetic rubber composites made with LGNR had similar dynamic mechanical properties to those made with NO, indicating that LGNR can act as alternative to traditional plasticizers for tire application. Replacing petroleum-based processing aids with LGNR can improve product performance and reduce the carbon footprint, increasing the sustainability of rubber products.

## 5. Patents

The work reported in this manuscript is under application for a patent (U.S. Patent App. 16/367,987).

## Figures and Tables

**Table 1:** Carbon-black-filled rubber compound compositions.

Material	phr								
HNR	100	100	100	0	0	0	0	0	0
SBR	0	0	0	100	100	100	0	0	0
GNR	0	0	0	0	0	0	100	100	100
Carbon black N330	50	50	50	50	50	50	50	50	50
Sulfur	4.5	4.5	4.5	4.5	4.5	4.5	4.5	4.5	4.5
ZnO	5	5	5	5	5	5	5	5	5
TBBS	1	1	1	1	1	1	1	1	1
Stearic acid	1	1	1	1	1	1	1	1	1
6PPD	2	2	2	2	2	2	2	2	2
NO	0	20	0	0	20	0	0	20	0
LGNR	0	0	20	0	0	20	0	0	20

Rubber compounds were made without processing aids (abbreviated as N/A is the text) or with 20 phr naphthenic oil (abbreviated as 20NO in the text) or with 20 phr liquid guayule natural rubber (abbreviated at 20LGNR in the text).

**Table 2:** Unfilled rubber compound composition.

Material	phr	
HNR	100	100
Sulfur	4.5	4.5
ZnO	5	5
TBBS	1	1
Stearic acid	1	1
6PPD	2	2
NO	0	10
LGNR	10	0

**Table 3:** Glass transition temperatures of naphthenic oil (NO) and liquid guayule natural rubber (LGNR). N/A: rubber compounds without processing aids; 20 NO: rubber compounds with 20 phr NO; 20 LGNR: rubber compounds with 20 phr LGNR.

Processing Aids	Glass Transition Temperature (°C)
NO	−54
LGNR	−62

**Table 4:** The tan δ of rubber composites at 0 and 60 °C. N/A: rubber compounds without processing aids; 20 NO: rubber compounds with 20 phr naphthenic oil; 20 LGNR: rubber compounds with 20 phr liquid guayule natural rubber.

Rubber Matrix	Processing Aids	tan δ at 0 °C	tan δ at 60 °C
HNR	N/A	0.21	0.12
HNR	20 NO	0.21	0.11
HNR	20 LGNR	0.24	0.16
SBR	N/A	0.21	0.09
SBR	20 NO	0.23	0.11
SBR	20 LGNR	0.21	0.12
GNR	N/A	0.23	0.16
GNR	20 NO	0.25	0.16
GNR	20 LGNR	0.26	0.18

## Data Availability

Not applicable.

## References

[1] Halász I.Z., Bárány T. (2015). Novel Bifunctional Additive for Rubbers: Cyclic Butylene Terephthalate Oligomer. Period. Polytech. Mech. Eng..

[2] Ortega L., Cerveny S., Sill C., Isitman N.A., Rodriguez-Garraza A.L., Meyer M., Westermann S., Schwartz G.A. (2019). The effect of vulcanization additives on the dielectric response of styrene-butadiene rubber compounds. Polymer.

[3] Azeem M., Borg-Karlson A.K., Rajarao G.K. (2013). Sustainable bio-production of styrene from forest waste. Bioresour. Technol..

[4] Priyadarshan P.M. (2011). Biology of Hevea Rubber.

[5] Priyadarshan P.M., Hoa T.T.T., Huasun H., Street T., Chi H., City M., Postal C. (2005). Yielding Potential of Rubber (*Hevea brasiliensis*) in Sub-Optimal Environments. J. Crop Improv..

[6] Salvucci M.E., Coffelt T.A., Cornish K. (2009). Improved methods for extraction and quantification of resin and rubber from guayule. Ind. Crops Prod..

[7] Premadasa R.B. ANRPC Releases Natural Rubber Trends & Statistics December 2020. http://www.anrpc.org/html/news-secretariat-details.aspx?ID=9&PID=39&NID=2271.

[8] GlobalData Global Styrene-Butadiene-Rubber (SBR) Industry Outlook to 2025—Capacity and Capital Expenditure Forecasts with Details of All Active and Planned Plants. https://store.globaldata.com/report/global-styrene-butadiene-rubber-sbr-industry-outlook-to-2025-capacity-and-capital-expenditure-forecasts-with-details-of-all-active-and-planned-plants/.

[9] Ramirez-Cadavid D.A., Cornish K., Michel F.C. (2017). Taraxacum kok-saghyz (TK): Compositional analysis of a feedstock for natural rubber and other bioproducts. Ind. Crops Prod..

[10] Ikeda Y., Junkong P., Ohashi T., Phakkeeree T., Sakaki Y., Tohsan A., Kohjiya S., Cornish K. (2016). Strain-induced crystallization behaviour of natural rubbers from guayule and rubber dandelion revealed by simultaneous time-resolved WAXD/tensile measurements: Indispensable function for sustainable resources. RSC Adv..

[11] Barrera C.S., Soboyejo A.B.O., Cornish K. (2017). Quantification of the Contribution of Filler Characteristics To Natural Rubber Reinforcement Using Principal Component Analysis. Rubber Chem. Technol..

[12] Barrera C.S., Cornish K. (2015). Novel Mineral and Organic Materials from Agro-Industrial Residues as Fillers for Natural Rubber. ASTM Int..

[13] Barrera C.S., Cornish K. (2017). Processing and mechanical properties of natural rubber/waste-derived nano filler composites compared to macro and micro filler composites. Ind. Crops Prod..

[14] Barrera C.S., Cornish K. (2016). High performance waste-derived filler/carbon black reinforced guayule natural rubber composites. Ind. Crops Prod..

[15] Rasutis D., Soratana K., McMahan C., Landis A.E. (2015). A sustainability review of domestic rubber from the guayule plant. Ind. Crops Prod..

[16] McMahan C., Kostyal D., Lhamo D., Cornish K. (2015). Protein influences on guayule and Hevea natural rubber sol and gel. J. Appl. Polym. Sci..

[17] Cornish K., Brichta J.L., Yu P., Wood D.F., Mcglothlin M.W., Martin J.A. (2002). Guayule latex provides a solution for the critical demands of the non-allergenic medical products market. Ind. Crop..

[18] Moore M. Ford, Cooper Make Inroads in Use of Guayule. http://www.rubbernews.com/article/20161123/NEWS/311149988/ford-cooper-make-inroads-in-use-of-guayule.

[19] Junkong P., Cornish K., Ikeda Y. (2017). Characteristics of mechanical properties of sulphur cross-linked guayule and dandelion natural rubbers. RSC Adv..

[20] Rensch G.J., Phillips P.J., Vatansever N., Gonzalez V.A. (1986). The crystallization behavior of Cis-polyisoprenes extracted from the guayule plant. J. Polym. Sci. Part B Polym. Phys..

[21] Li J., Isayev A.I., Ren X., Soucek M.D. (2016). Toward Replacement of Petroleum Oils By Modified Soybean Oils in Elastomers. Rubber Chem. Technol..

[22] Petrović Z.S., Ionescu M., Milić J., Halladay J.R. (2013). Soybean Oil Plasticizers As Replacement of Petroleum Oil in Rubber. Rubber Chem. Technol..

[23] Wang H., Zhuang T., Shi X., Van Duin M., Zhao S. (2018). Peroxide cross-linking of EPDM using moving die rheometer measurements. II: Effects of the process oils. RUBBER Chem. Technol..

[24] (2005). EU Directive 2005/69/EC of the European Parliament and of the Council of 16 November 2005 amending for the 27th time Council Directive 76/769/EEC on the approximation of the laws, regulations and administrative provisions of the Member States relating to res. Off. J. Eur. Union.

[25] Menon A.R.R., Pillai C.K.S., Nando G.B. (1999). Cure characteristics and physicomechanical properties of natural rubber modified with phosphorylated cashew nut shell liquid prepolymer-A comparison with aromatic oil. J. Appl. Polym. Sci..

[26] Wang Z., Peng Y., Zhang L., Zhao Y., Vyzhimov R., Tan T., Fong H. (2016). Investigation of Palm Oil as Green Plasticizer on the Processing and Mechanical Properties of Ethylene Propylene Diene Monomer Rubber. Ind. Eng. Chem. Res..

[27] Raju P., Nandanan V., Kutty S.K.N. (2007). A Study on the Use of Castor Oil as Plasticizer in Natural Rubber Compounds. J. Rubber Res..

[28] Nandanan V., Joseph R., Francis D.J. (1996). Linseed Oil as a Multipurpose Ingredient in NBR Vulcanizate. J. Elastomers Plast..

[29] Saramolee P., Sahakaro K., Lopattananon N., Dierkes W.K., Noordermeer J.W.M. (2014). Comparative properties of silica-and carbon black-reinforced natural rubber in the presence of epoxidized low molecular weight polymer. Rubber Chem. Technol..

[30] Ren X., Barrera C.S., Tardiff J.L., Gil A., Cornish K. (2020). Liquid guayule natural rubber, a renewable and crosslinkable processing aid in natural and synthetic rubber compounds. J. Clean. Prod..

[31] Cornish K. (1996). Hypoallergenic Natural Rubber Products from *Parthenum argentatum* (Gray) and Other Non-Hevea Brasiliensis Species. US Patent.

[32] Wang M. (1998). Effect of polymer-filler and filler-filler interactions on dynamic properties of filled vulcanizates. Rubber Chem. Technol..

[33] Mohanty T.R., Bhandari V., Chandra A.K., Chattopadhyay P.K., Chattopadhyay S. (2013). Role of calcium stearate as a dispersion promoter for new generation carbon black-organoclay based rubber nanocomposites for tyre application. Polym. Compos..

[34] Ren X., Sancaktar E. (2019). Use of fly ash as eco-friendly filler in synthetic rubber for tire applications. J. Clean. Prod..

[35] Flory P.J., Rehner J. (1943). Statistical Mechanics of Cross-Linked Polymer Networks I. Rubberlike Elasticity. J. Chem. Phys..

[36] (2014). ASTM Standard D6814−02 Standard Test Method for Determination of Percent Devulcanization of Crumb Rubber Based on Crosslink Density. ASTM Int..

[37] Marzocca A.J. (2007). Evaluation of the polymer–solvent interaction parameter X for the system cured styrene butadiene rubber and toluene. Eur. Polym. J..

[38] Kulkarni P.P., Jafvert C.T. (2008). Solubility of C_60_ in Solvent Mixtures. Environ. Sci. Technol..

[39] Thaijaroen W. (2011). Effect of tackifiers on mechanical and dynamic properties of carbon-black-filled NR vulcanizates. Polym. Eng. Sci..

[40] Pazur R.J., Kennedy T.A.C. (2015). Effect of Plasticizer Extraction By Jet Fuel on a Nitrile Hose Compound. Rubber Chem. Technol..

[41] Rattanasom N., Saowapark T., Deeprasertkul C. (2007). Reinforcement of natural rubber with silica/carbon black hybrid filler. Polym. Test..

[42] Choi S.-S. (2000). Influence of rubber composition on change of crosslink density of rubber vulcanizates with EV cure system by thermal aging. J. Appl. Polym. Sci..

[43] Joseph A.M., George B., Madhusoodanan K.N., Alex R. (2015). Current status of sulphur vulcanization and devulcanization chemistry: Process of vulcanization. Rubber Sci..

[44] Zhao J., Yang R., Iervolino R., Barbera S. (2013). Changes of Chemical Structure and Mechanical Property Levels During Thermo-Oxidative Aging of Nbr. Rubber Chem. Technol..

[45] Hamed G.R., Zhao J. (1999). Tensile Behavior after Oxidative Aging of Gum and Black-Filled Vulcanizates of SBR and NR. Rubber Chem. Technol..

